# The Composition of the Microbiota in the Full-Term Fetal Gut and Amniotic Fluid: A Bovine Cesarean Section Study

**DOI:** 10.3389/fmicb.2021.626421

**Published:** 2021-04-30

**Authors:** Aleksi Husso, Leen Lietaer, Tiina Pessa-Morikawa, Thomas Grönthal, Jan Govaere, Ann Van Soom, Antti Iivanainen, Geert Opsomer, Mikael Niku

**Affiliations:** ^1^Department of Veterinary Biosciences, Faculty of Veterinary Medicine, University of Helsinki, Helsinki, Finland; ^2^Department of Reproduction, Obstetrics and Herd Health, Faculty of Veterinary Medicine, Ghent University, Merelbeke, Belgium; ^3^Central Laboratory, Faculty of Veterinary Medicine, University of Helsinki, Helsinki, Finland

**Keywords:** bovine, gut, amniotic fluid, meconium, fetal, cesarean section, microbiome, neonatal

## Abstract

The development of a healthy intestinal immune system requires early microbial exposure. However, it remains unclear whether microbial exposure already begins at the prenatal stage. Analysis of such low microbial biomass environments are challenging due to contamination issues. The aims of the current study were to assess the bacterial load and characterize the bacterial composition of the amniotic fluid and meconium of full-term calves, leading to a better knowledge of prenatal bacterial seeding of the fetal intestine. Amniotic fluid and rectal meconium samples were collected during and immediately after elective cesarean section, performed in 25 Belgian Blue cow-calf couples. The samples were analyzed by qPCR, bacterial culture using GAM agar and 16S rRNA gene amplicon sequencing. To minimize the effects of contaminants, we included multiple technical controls and stringently filtered the 16S rRNA gene sequencing data to exclude putative contaminant sequences. The meconium samples contained a significantly higher amount of bacterial DNA than the negative controls and 5 of 24 samples contained culturable bacteria. In the amniotic fluid, the amount of bacterial DNA was not significantly different from the negative controls and all samples were culture negative. Bacterial sequences were identified in both sample types and were primarily of phyla Proteobacteria, Firmicutes, Bacteroidetes, and Actinobacteria, with some individual variation. We conclude that most calves encounter *in utero* maternal-fetal transmission of bacterial DNA, but the amount of bacterial DNA is low and viable bacteria are rare.

## Introduction

Characterizing the very first intestinal bacteria is essential for a better understanding of the co-development of the newborn and its intestinal microbiome. Host-microbiome interactions enable early life education and maturation of the immune system during a *window of opportunity*. The use of prenatal and intrapartum antibiotics may disturb this process (Dierikx et al., 2020). However, the timing of the microbial colonization of the mammalian gut is still unclear (Perez-Munoz et al., 2017; Korpela and de Vos, 2018; Liu et al., 2019; Guzman et al., 2020; Blaser et al., 2021; Silverstein and Mysorekar, 2021). Vertical as well as environmental transmission of bacteria occur during and after birth and seed the neonatal gastrointestinal (GI) tract (Funkhouser and Bordenstein, 2013; Gomez de Agüero et al., 2016; Korpela and de Vos, 2018). Transmission of an orally inoculated *Enterococcus faecium* strain in pregnant mice to the meconium of their fetuses has been described (Jimenez et al., 2008).

Recent studies in humans, mice and cattle have reported the identification of microbial DNA or culturable bacteria in the mammalian fetal environment (Aagaard et al., 2014; Seferovic et al., 2019; Al Alam et al., 2020; Guzman et al., 2020; Rackaityte et al., 2020). Nevertheless, others interpret such observations as intrauterine infections or contamination (Lauder et al., 2016; Eisenhofer et al., 2018; de Goffau et al., 2019; Hockney et al., 2020; Olomu et al., 2020; Theis et al., 2020a,b). The newborn’s GI tract is a low microbial biomass environment, implying multiple challenges for performing reliable microbiome analyses (Glassing et al., 2016; Eisenhofer et al., 2018; Stinson et al., 2018). Besides the technical limitations inherent to DNA sequencing, there are practical and ethical concerns while collecting samples in humans. First-pass meconium has been used as a proxy to assess the fetal intestinal microbiome, although samples are often collected hours after birth and even breastfeeding, suggesting potential postnatal effects on the microbial composition of the meconium (Hansen et al., 2015; Collado et al., 2016; Liu et al., 2019; Stinson et al., 2019). Mammalian animal models can overcome several of these ethical and practical difficulties, providing insights in the fetal gastrointestinal microbiome.

In the current study, the microbial composition of the full-term fetal gut and the corresponding amniotic fluid was assessed in Belgian Blue cow-calf couples. In this double-muscled beef breed, cesarean sections (C-sections) are performed on a routine basis during the very early stages of parturition, while fetal membranes are still intact, rendering this breed a highly suitable model for full-term gestation microbiome studies.

Besides the potential role of the cow model for research, there is also a significant interest in the composition and the development of the calf’s intestinal microbiome. Gut health and growth performance during the first weeks of life are main drivers for cost effective livestock rearing (Thornton, 2010; Lorenz et al., 2011). Few studies have been conducted on the intestinal microbiome in vaginally born neonatal calves. These have reported low numbers of bacteria, mostly belonging to the phyla Proteobacteria, Firmicutes, Actinobacteria, and Bacteroidetes (Alipour et al., 2018; Klein-Jöbstl et al., 2019; Guzman et al., 2020). Shared microbiota were found between calf meconium and the maternal vaginal vestibulum (Alipour et al., 2018; Yeoman et al., 2018; Klein-Jöbstl et al., 2019). To the best of our knowledge, no microbiome studies have yet been performed on calves born by C-section, and no studies are available to assess the association between the microbial DNA signatures in meconium and the corresponding amniotic fluid.

Our aims were to assess the bacterial load and characterize the bacterial composition of the amniotic fluid and meconium of full-term neonatal calves. To this end, samples were collected during elective C-section, and analyzed by qPCR, bacterial culture under carefully controlled and validated conditions, and 16S rRNA gene amplicon sequencing. To minimize reagent and environmental bacterial DNA contaminants, we included multiple technical controls and stringently filtered the 16S rRNA gene sequencing data to exclude putative contaminant sequences.

## Materials and Methods

All experimental procedures were approved by the institutional ethics and animal welfare committee of the Faculty of Veterinary Medicine (EC2018/002 - Ghent University, Belgium). The cows’ owners were informed about the study and gave their written consent.

### Study Design

The sampling of this study was performed at the teaching hospital of the Department of Reproduction, Obstetrics, and Herd Health of the Faculty of Veterinary Medicine in Ghent (Belgium), where pregnant Belgian Blue beef cows of different herds (parity between 1 and 5) were housed for an elective C-section.

In total 25 Belgian Blue cows and their calves (7 males and 18 females) were sampled from November 2017 until March 2019. The cows were housed in tie-stalls at the facility for 9.5 d ± 5.8 (mean ± standard deviation) prior to C-section and had *ad libitum* access to hay and water. During this period, rectal temperature was measured twice daily, and calving indicators such as udder distension, teat filling, pelvic ligament relaxation, vaginal discharge, vulvar edema, and behavioral changes were monitored every 2 h by graduate veterinary students.

Prior to elective C-section, in cows that exhibited a drop in temperature, cervical dilation was assessed by manual palpation. The vulvar region was cleaned with iodine soap and water. A gloved hand was inserted vaginally and the opening of the portio vaginalis cervicis was estimated. For the present study, elective C-section was performed when the cow had a minimal cervical dilation of 8 cm, with no rupture of the fetal membranes prior to surgery. All cows were healthy according to their vital parameters (heart rate, temperature, respiratory rate) and there was no clinical evidence of intrauterine infection or contamination.

### Sampling

Prior to surgery, the cows were restrained in a standing position in a surgery chute specifically designed for cattle. C-section procedure was done as described by Kolkman et al. (2007). Briefly, the surgical area (left flank) was washed and disinfected, an abdominal incision was made, and part of the uterus was exteriorized for uterotomy. The allantoic sac was opened up to expose the intact amniotic sac. Amniotic fluid was aspirated through the amniotic membrane, using a sterile 16 G needle (Agani, Terumo Europe, Hamburg, Germany) and sterile 20 ml syringe (B. Braun, Melsungen, Germany). Within 1 h after sampling, the retrieved volume was aliquoted, under a laminar-flow hood, into 2 sterile 15 ml tubes (188271, Cellstar, Greiner bio-one, Frickenhausen, Germany). In the first tube, 6 ml of amniotic fluid was dissolved in 3 ml of glycerol (≥99%, G2025, Sigma-Aldrich (Merck), Overijse, Belgium) and subsequently stored at −80°C to be used in culture experiments. In the second tube, 12 ml of amniotic fluid was stored at −80°C to be used for bacterial DNA extraction.

Meconium samples were acquired directly from the calves’ rectum, immediately after birth (no more than 30 min). Until the moment of sampling, calves laid on a clean concrete floor, with access to neither the dam, nor colostrum. The perineum of the calf was dried with a clean paper towel and disinfected with 70% ethanol. A sterile double-guarded equine uterine culture swab (Har-vet, 17705, Spring Valley, United States; or Minitube, 17214/2950, Tiefenbach, Germany) was gently introduced in the rectum and the swab was exposed. Samples were taken in duplicate, one stored immediately at −80°C with no additives, and the other stored at −80°C in a sterile 2 ml cryovial containing 1 ml of a 30% glycerol solution, prepared by diluting glycerol (≥99%, G2025, Sigma-Aldrich (Merck), Overijse, Belgium), in ultra-pure, nuclease-free water (W4502, Sigma-Aldrich (Merck), Overijse, Belgium) to a final 30% concentration.

Negative field controls were processed in the surgery room, using the same sampling procedures and disposables. In total, 16 empty, sterile double-guarded equine uterine culture swabs (10 Har-vet swabs and 6 Minitube swabs) were included for the meconium sampling, 5 of the Har-vet swabs stored in 1 ml of a 30% glycerol stock solution, the others with no additives. Additionally, 12 negative field controls were included for the amniotic fluid sampling, aspirating ultra-pure, nuclease-free water (W4502, Sigma-Aldrich (Merck), Overijse, Belgium) instead of amniotic fluid. For 8 of them, the ultra-pure, nuclease-free water was stored with no additives, and for 4 of them, 6 ml ultra-pure, nuclease-free water was dissolved in 3 ml of glycerol.

All samples were shipped on dry ice to the laboratory of the Department of Veterinary Biosciences of the Faculty of Veterinary Medicine in Helsinki (Finland) for further processing.

### Culture

#### Validation of Culture Media

We aimed to define a single bacterial culture medium, capable of sustaining a majority of the calf intestinal core microbiota. Consequently, this single medium could be used for culturing the meconium and amniotic fluid samples, avoiding further splitting or dilution of the low biomass samples. We tested GAM “Nissui” medium (Gifu Anaerobic Medium Agar, Code 05420, HyServe, Germany) mostly used for anaerobic bacteria, YCFA medium (Yeast extract, Casitone and Fatty Acid) containing volatile fatty acids typically supporting the growth of several gut bacteria, LB medium (Lysogeny Broth, BD) as a general medium and BB medium (Trypticase Soy Agar supplemented with Bovine Blood 211043, Tammer BioLab Oy, Tampere, Finland) supporting fastidious bacteria by blood enrichment. Each medium was tested for the ability to sustain calf intestinal core microbiota, by plating each type anaerobically with feces of a healthy 7 days old calf (Lopez-Siles et al., 2012; Alipour et al., 2018). After 7 days of growth, mixed cultures were extracted from the plates, 16S rRNA gene amplicon sequenced, and compared to the core microbiota previously observed in fecal samples of young calves (Alipour et al., 2018).

#### Sample Culturing

Bacterial culture on GAM-agar plates was performed for 24 meconium samples (5 corresponding negative field controls) and 24 amnion samples (4 negative field controls). The frozen samples were first transferred to an anaerobic workstation (Ruskinn Concept Plus), mixed thoroughly and plated, using an aseptic technique, at +37°C. After this, the samples were transferred to a laminar flow cabinet and plated in aerobic conditions at +37°C. The culture plates were checked for growth daily for 14 days. All visible bacterial colonies were subcultured until pure isolates were obtained. Fresh cultures were then identified using MALDI-TOF (Bruker Microflex LT) at the Central laboratory of the Faculty of Veterinary Medicine (Helsinki, Finland).

Samples were prepared using MALDI Biotyper MSP Identification Standard Method v 1.1. Mass spectra were analyzed in a mass/charge range from 2,000 to 20,000 Da with MBT Compass v4.1 on flexControl v3.4 (Bruker Daltonik GmbH) using BDAL-7311 as the reference library. The Bruker Bacterial Test Standard (RUO) (Bruker Daltonik GmbH) was used for instrument calibration. If the identification confidence score was < 2.00, further identification was done with 16S rRNA gene amplicon Sanger sequencing at the Institute of Biotechnology (University of Helsinki, Finland).

### DNA Extraction

DNA from the meconium samples (*N* = 25) and corresponding negative field controls (*N* = 11) were extracted using ZymoBIOMICS DNA Miniprep Kit (Zymo Research, Irvine, CA, United States) according to the manufacturer’s instructions, with minor modifications to the protocol as described previously (Alipour et al., 2018; Husso et al., 2020). ZymoBIOMICS^TM^ Microbial Community Standard and an in-house fecal standard were processed with the meconium samples in every batch.

For the amniotic fluid samples (*N* = 23) and the corresponding negative field controls (*N* = 8), 2 ml of each sample was first centrifuged in a microcentrifuge at 16,100 × g 10 min in +4°C. Most of the supernatant was removed and 750 μl of ZymoBIOMICS Lysis Solution and 19 μl of proteinase K (D3001-2-20/D3001-2-5, Zymo Research) were added to the remaining 200 μl of the samples and incubated for 30 min at +55°C. After these steps, the same protocol as applied for the meconium samples was followed.

All manipulations of the tubes during the process were performed in a laminar flow cabinet, and the workplace, instruments and pipettes were cleaned routinely with 10% bleach. Certified DNA, RNase, DNase and PCR inhibitor free tubes (STARLAB International, Germany) and Nuclease-free Water (Ambion, Thermo Fisher Scientific, United States) were used for DNA extraction and downstream analyses. All extracted DNA was stored at −80°C.

### Quantitative PCR

The bacterial 16S rRNA gene copy numbers in the meconium, amnion and negative field control samples were determined using quantitative PCR. The analyses were performed as described previously (Alipour et al., 2018; Husso et al., 2020), with the exception that the PCR master mix was first treated using a dsDNAse based decontamination kit (Enzo Life Sciences, Farmingdale, New York). The amplification was performed using the Bio-Rad CFX96 instrument (Bio-Rad, Hercules, California) and universal eubacteria probe and primers (Nadkarni et al., 2002). A standard series, negative field controls and no-template controls were included in every run. The data were analyzed using the Bio-Rad CFX Maestro software.

### Library Preparation and 16S rRNA Gene Amplicon Sequencing

The V3-V4 region of the 16S rRNA gene amplicons was sequenced using the Illumina MiSeq platform in the DNA core facility of the University of Helsinki, as described previously (Alipour et al., 2018; Husso et al., 2020). In total, 23 amniotic fluid samples and 23 meconium samples of the same cow-calf couple were sequenced, together with the corresponding field controls, no-template controls, a ZymoBIOMICS Microbial Community Standard (Zymo Research, United States) and an in-house adult cow fecal standard. The observed composition and abundances for the commercial standard matched the expected composition provided by the manufacturer (data not shown).

The numbers of pre-amplification PCR cycles were optimized based on the amounts of bacterial DNA template in each sample type. The ZymoBIOMICS Microbial Community Standard and the in-house adult cow fecal standard were pre-amplified with 12 cycles and all other sample types, including negative controls, with 21 cycles.

### Bioinformatics

The detailed bioinformatics pipeline is described in Supplementary Materials. Briefly, the read quality was first inspected with FastQC and MultiQC (Andrews, 2010; Ewels et al., 2016). Leftover primes and spacers were then trimmed with Cutadapt v1.10 (Martin, 2011). A mapping file was created for QIIME2 and validated with Keemei (Rideout et al., 2016). The FASTQ-files were imported to QIIME2 v2019.4, where the DADA2 plugin was used to denoise and quality filter the reads, call ASVs and generate a feature table (Callahan et al., 2016; Bolyen et al., 2019). A naïve Bayes classifier was trained in QIIME2 against SILVA v132 99% database, extracted to only include the V3-V4 region and used to assign taxonomy to ASVs (Quast et al., 2013; Bokulich et al., 2018). Sequences derived from chloroplasts or mitochondria were removed and singletons were filtered out, leaving only bacteria with at least phylum-level identification.

### Decontamination of the 16S rRNA Gene Amplicon Sequencing Data

The processed data was *in silico* filtered to remove amplicon sequence variants (ASVs) which represented probable contaminants (reagent contaminants and environmental bacterial DNA), as described previously (Husso et al., 2020).

Briefly, an ASV was removed if its prevalence in actual samples was ≤2× its prevalence in field controls (DNA extracted from empty sampling instruments exposed to the surgery room environment), and if its mean relative abundance in actual samples was ≤ 10 × its mean abundance in field controls (Supplementary Figure 1). The filtering was performed separately for meconium and amnion samples. If less than 500 reads remained after the decontamination, as was the case for six meconium samples, the sample was removed from further analyses. The original and remaining read counts are shown in Table 1.

**TABLE 1:** Mean read counts (standard deviation) detected in meconium and amniotic fluid samples and their negative controls.

**Sample type**	**Raw**		**Processed**		**Decontaminated**	
	**N**	**Reads**	**N**	**Reads**	**N**	**Reads**
Meconium	23	92,266 (36,235)	23	57,082 (25,543)	17	4,189 (8,931)
Meconium control	11	126,800 (21,164)	11	84,023 (14,585)	–	–
Amnion	23	127,797 (22,376)	23	83,279 (15,120)	23	3,338 (2,922)
Amnion control	6	136,488 (12,663)	6	90,802 (8,998)	–	–

*Only sequences with at least phylum-level identification were retained for further analyses. If less than 500 reads remained after in silico decontamination, as in six meconium samples, the sample was removed from further analyses.*

### Statistics

The 16S rRNA gene qPCR results from samples and negative field controls were compared using two-tailed Mann-Whitney U test in IBM SPSS Statistics 25. Shannon diversity indices were calculated for genus-level data with the R package phyloseq without rarefaction (McMurdie and Holmes, 2013). The Kruskal-Wallis rank sum test was used to compare the bacterial community structure of both sample types in RStudio (R Studio Team, 2020). The PCoA figures were plotted using ASV and genus-level data and Bray-Curtis distances with the R package phyloseq (McMurdie and Holmes, 2013). Permutational analysis of variance (PERMANOVA) and multivariate homogeneity of group dispersions (betadisper) were calculated using the R package vegan, using 9,999 permutations (Oksanen et al., 2019). DESeq2 was used to explore differentially abundant ASVs by calculating differential expression between sample groups (Love et al., 2014). The LEfSe (Linear discriminant analysis Effect Size) web application was used to identify the taxons most likely explaining the differences between the sample types (Segata et al., 2011). An ecologically organized heatmap of the top 40 most abundant ASVs was created with the R package phyloseq (Rajaram and Oono, 2010; McMurdie and Holmes, 2013). Spearman correlations and their Bonferroni corrected *p*-values were calculated using R-package Hmisc 4.3.0 (Harrel, 2019), using the absolute counts of ASVs and genera that were present >100 times in all of the samples. Differences were considered significant at *P* < 0.05.

## Results

### Quantification of Bacterial 16S rRNA Genes in Amniotic Fluid and Meconium Samples by qPCR

**FIGURE 1:**
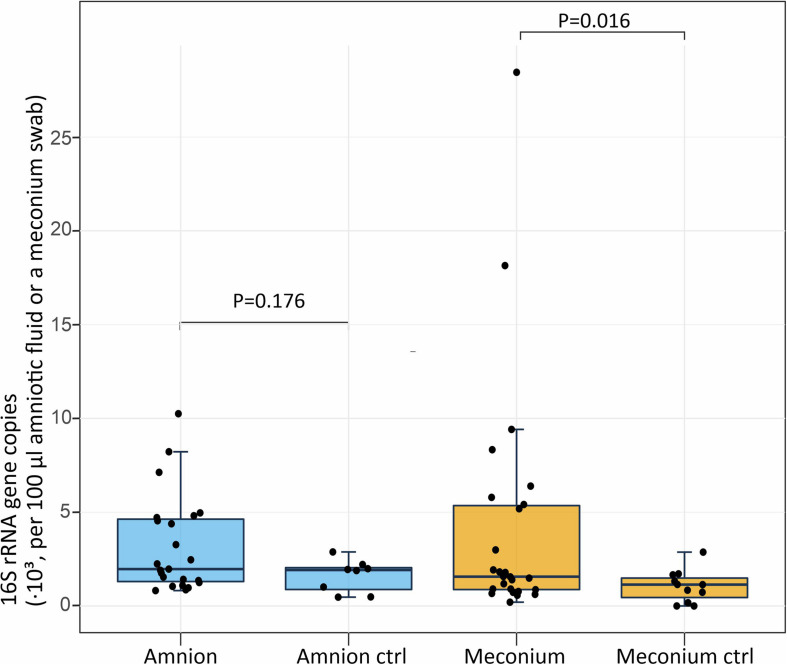
Absolute 16S rRNA gene copy numbers per 100 μl of centrifuged amniotic fluid (*n* = 23, negative controls *n* = 8) and per meconium sampling swab (*n* = 25, negative controls *n* = 11). The midline of the box is the median, with the upper and lower limits of the box being the third and first quartile. The whiskers extend up to 1.5 times the interquartile range from the box to the furthest data point within that distance.

The 16S rRNA gene copy numbers in amniotic fluid and meconium samples were assessed by qPCR (Figure 1). In the amniotic fluid samples, there was no significant difference (*P* = 0.176) between the 16S rRNA gene copy numbers of the samples (*N* = 23; mean = 3,170 copies per 100 μl of centrifuged amniotic fluid; *SD* = 2,580) and the negative field controls (*N* = 8; mean = 1,610; *SD* = 790). In the meconium samples, the 16S rRNA gene copy number (*N* = 25; mean = 4,350 copies per sampling swab; *SD* = 6,410) was significantly higher (*P* = 0.016) than in the negative field controls (*N* = 11; mean = 980; *SD* = 850).

### Microbial Composition of Amniotic Fluid and Meconium Samples

**FIGURE 2:**
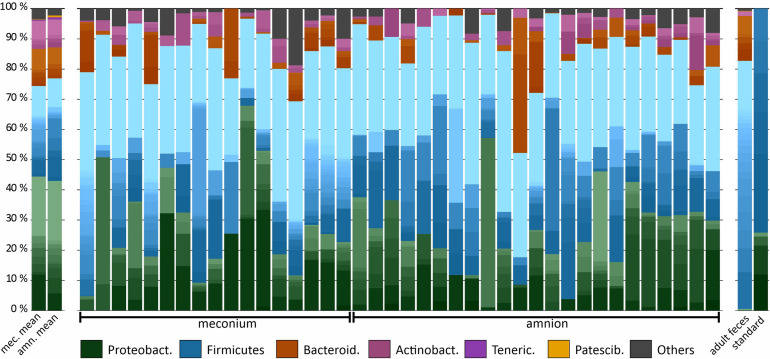
Microbiota composition in bovine meconium and amniotic fluid samples collected during elective C-section, adult feces, and a commercial community composition standard. The main colors indicate the bacterial phyla. Within phyla, the shades indicate bacterial genera. The lightest shade of each phylum shows the combined abundance of the least abundant genera (with a maximum of < 0.5% of total).

**FIGURE 3:**
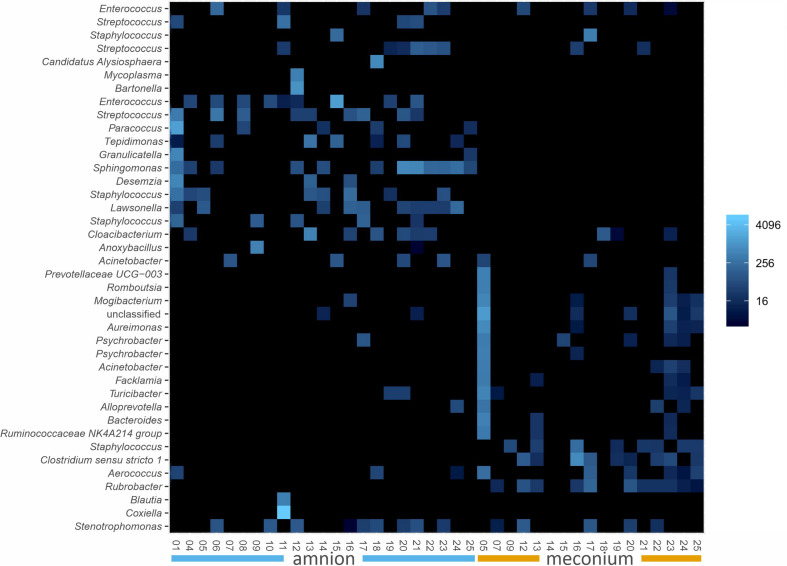
Ecologically organized heatmap (NMDS, Bray) of the 40 most abundant ASVs in bovine meconium and amniotic fluid samples collected during elective C-section, sorted by sample type. The taxon names are presented as genus level identifications. Color scale indicates relative abundance and is a log transformation with base 4.

We analyzed the microbial DNA profiles in the fetal samples using 16S rRNA gene amplicon sequencing. The microbial signature of both sample types consisted primarily of Proteobacteria, Firmicutes, Bacteroidetes, and Actinobacteria phyla, with individual variation (Figure 2). A heatmap analysis of the 40 most abundant ASVs shows the difference between the sample types in more detail (Figure 3). In both sample types, the inter-individual variation increased at lower taxonomic levels (Supplementary Table 1 and Figure 3). The most abundant bacterial genera in meconium were *Delftia*, *Staphylococcus*, and *Clostridium sensu stricto 1*, while the most prevalent genera were *Delftia*, *Acinetobacter*, unclassified *Burkholderiaceae*, *Staphylococcus*, and *Corynebacterium 1* (Supplementary Table 1). In the amniotic fluid samples, *Staphylococcus*, *Streptococcus, Delftia, Sphingomonas*, and *Enterococcus* were the most abundant genera, and *Delftia*, *Streptococcus*, *Staphylococcus, Sphingomonas*, and *Acinetobacter* were the most prevalent genera (Supplementary Table 1).

**FIGURE 4:**
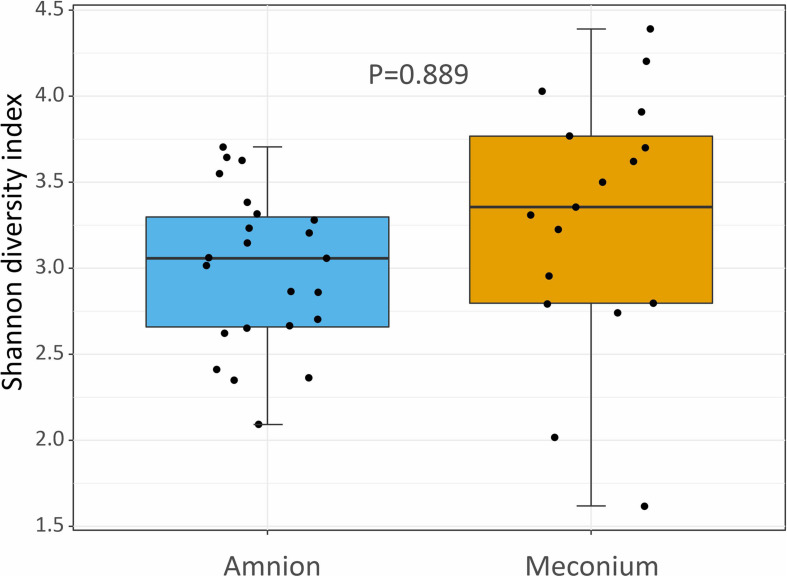
Shannon diversity index showing the bacterial community structure (at genus level) of amniotic fluid and meconium samples, collected during elective C-section in Belgian Blue cow-calf pairs. Boxplot as in Figure 1.

The alpha diversity (Shannon index) at the genus level was not significantly different between the meconium and amniotic fluid samples (Figure 4, *P* = 0.889).

### Comparison of Amniotic Fluid and Meconium Microbial DNA Signature

**FIGURE 5:**
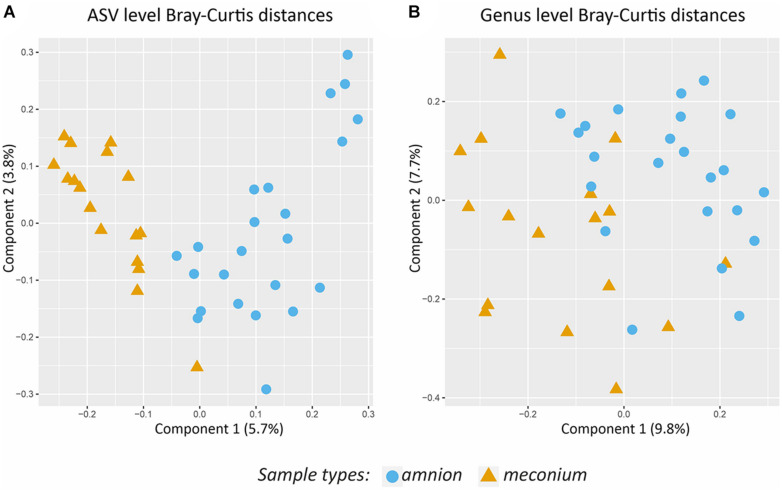
Comparison of the bacterial community structure of amniotic fluid and meconium. **(A)** PCoA on Bray-Curtis distances based on ASV level data. **(B)** PCoA on Bray- Curtis distances based on genus level data. Colors and shapes indicate the sample types.

The microbial DNA signature was significantly different for amniotic fluid and meconium samples at the ASV level (PERMANOVA, *P* < 0.001, *R*^2^ = 0.049, Figure 5A), and genus level (PERMANOVA, *P* < 0.001, *R*^2^ = 0.062, Figure 5B).

According to the DESeq2 analysis, three ASVs were more abundant in meconium than in amniotic fluid: one *Staphylococcus* ASV (log2 Fold Change = 25.084, *P*_adj_ < 0.001), one *Rubrobacter* ASV (log2 Fold Change = 8.337, *P*_adj_ < 0.001), and one *Clostridium* ASV (log2 Fold Change = 8.055, *P*_adj_ = 0.004). In amniotic fluid, one *Sphingomonas* ASV (log2 Fold Change = −8.420, *P*_adj_ < 0.001) and one *Staphylococcus* ASV (log2 Fold Change = −23.438, *P*_adj_ < 0.001) were more abundant than in meconium.

By LefSe (Linear discriminant analysis effect size) analysis including all taxonomic levels, meconium samples had a greater relative abundance of *Clostridiales* and *Rubrobacter* (LDA score (log10) > 4.0) than the amniotic fluid, but a lower relative abundance of Bacillales and *Corynebacteriales* (LDA score (log10) < −4.0).

### Correlations Between Meconium and Amniotic Fluid From the Same Animal

To assess whether the meconium and amniotic fluid microbial DNA profiles may have common origins, we calculated Spearman rank correlations between meconium and amniotic fluid 16S rRNA gene sequencing data, using the absolute counts of ASVs and genera that were present > 100 times in all of the samples. Average correlations (ρ) at ASV (*N* = 17, ρ_avg_ = −0.0201, *P*_avg_ < 0.05) and genus level (*N* = 17, ρ_avg_ = 0.1685, *P*_avg_ < 0.05) were very weak and biologically not significant. All *p*-values were Bonferroni corrected for multiple testing.

### Bacterial Cultures

Bacterial culture was performed to assess the presence of viable bacteria in the fetal samples. Based on our testing of multiple media, the GAM (Gifu Anaerobic Medium) agar sustained the largest number of bacterial core genera found in calf feces (Alipour et al., 2018), including some additional unique genera not found on any other plate type. Thus, we selected this medium for these experiments.

All amniotic samples and their negative field controls were culture negative, both in anaerobic and aerobic conditions. Five out of 24 meconium samples and 1 out of 5 negative field controls were culture positive on GAM agar. The results for each sample are presented in detail in Table 2. Both gram-negative and gram-positive strains were isolated from the samples.

**TABLE 2:** Culture positive meconium samples and identified bacteria.

**Sample**	**Aerobic strains**	**Anaerobic strains**
Negative control 1	*Staphylococcus* sp. (Gram+), *Kocuria* sp. (Gram+)	*Rothia* sp. (Gram+)
Meconium 5	*Roseomonas* sp. (Gram−)	–
Meconium 10	*Acinetobacter* sp. (Gram−)	*Streptococcus* sp. (Gram+), *Fusobacterium* sp. (Gram−)
Meconium 12	–	*Cutibacterium* sp. (Gram+)
Meconium 16	*Kocuria* sp. (Gram+)	–
Meconium 21	*Achromobacter* sp. (Gram−)	–

*If the identification confidence score was < 2.00 for MALDI-TOF, further identification was done with 16S rRNA gene amplicon Sanger sequencing to achieve genus level identification for all isolates.*

The Sanger sequence from the *Roseomonas* sp. isolate acquired from a meconium sample was a 100% identical match to the 16S rRNA gene amplicon sequencing data from the same sample. In addition, the Sanger sequences of *Staphylococcus* sp., *Kocuria* sp., *Roseomonas* sp., and *Acinetobacter* sp. isolates matched the 16S rRNA gene amplicon sequencing data when compared to all meconium samples. Three bacterial genera could be identified in the negative controls: *Rothia* sp., *Staphylococcus* sp., and *Kocuria* sp. Of these, only *Kocuria* sp. was also identified from the actual meconium samples.

## Discussion

Vertical transmission of microbiota *in utero* remains controversial (Korpela and de Vos, 2018; Liu et al., 2019; Blaser et al., 2021; Silverstein and Mysorekar, 2021). In this study, we used a carefully controlled bovine elective C-section model for assessing the bacterial load and microbiota composition in full-term fetal gut and amniotic fluid.

We first examined the presence of microbial DNA in meconium and amniotic fluid by 16S rRNA gene qPCR and amplicon sequencing. Since contamination is a major challenge in the analysis of low bacterial biomass samples, we processed several types of negative controls alongside our biological samples, treated the qPCR mastermix with dsDNAse, and applied rigorous *in silico* filtering of potential contaminants (Glassing et al., 2016; Eisenhofer et al., 2018).

We observed a small but significant amount of bacterial DNA in the meconium samples by 16S qPCR, suggesting prenatal transmission of bacterial DNA to the fetal intestine. This is consistent with previous studies in cattle, in which vaginally born calves were sampled (Mayer et al., 2012; Alipour et al., 2018; Yeoman et al., 2018; Klein-Jöbstl et al., 2019). A profile dominated by the phyla Proteobacteria, Firmicutes, Bacteroidetes, and Actinobacteria was identified in the meconium, in agreement with previous studies in newborn animals or slaughterhouse fetuses (Alipour et al., 2018; Yeoman et al., 2018; Klein-Jöbstl et al., 2019; Guzman et al., 2020; Husso et al., 2020). In contrast, Malmuthuge and Griebel (2018) concluded that fetal ovine intestinal tissue collected in C-sections were devoid of any bacterial DNA. Their PCR and agarose gel protocols were likely not sufficiently sensitive to detect small amounts of specific template as all the amplifications appeared negative, although probe-based qPCR is generally able to detect contaminating microbial DNA even in high-quality molecular biology reagents. Such technical differences may explain the different conclusions from 16S rRNA gene amplicon sequencing. Malmuthuge and Griebel (2018) obtained an extremely small number of reads also from their positive controls and compared the samples and controls only at phylum level.

In amniotic fluid, the amount of bacterial DNA was insignificant. However, we observed a 16S rRNA gene sequence profile distinguishable from the negative controls, suggesting the presence of microbial DNA at a very low abundance. Previous studies on amniotic fluid microbiota have yielded conflicting results and the study designs are diverse further complicating their comparisons. The amniotic fluid sampled by Collado et al. (2016) in women during pre-labor C-section was found to contain a distinct, low diversity, low biomass microbiome, predominated by Proteobacteria (*Enterobacteriaceae*). Malmuthuge and Griebel (2018) did not observe bacteria in their ovine amniotic fluid samples, within technical limits described above. Moore et al. (2017) reported a microbiome in bovine amniotic fluid by 16S rRNA gene amplicon sequencing, but did not utilize sufficient negative controls to accurately study low-abundance microbiota. Guzman et al. (2020) observed bacterial DNA in bovine amniotic fluid by qPCR and sequencing. The microbial DNA signature was similar to the one in our study at the phylum level but differed at family and genus levels.

We observed 16s rRNA genes of several intestine associated bacterial taxa in the meconium and/or amniotic fluid samples, including *Bacteroides*, *Clostridium*, *Enterococcus*, Lachnospiraceae, Christensenellaceae, and Ruminococcaceae. These most likely originate from the dam. Some of the other commonly mucosa-associated genera, such as *Lactobacillus, Streptococcus, Staphylococcus*, and *Corynebacterium* are also prevalent in human meconium (Chu et al., 2017). *Sphingomonas*, *Acinetobacter, Pseudomonas*, and *Stenotrophomonas* were also previously described in neonatal calves (Klein-Jöbstl et al., 2019). Some of the prevalent genera found in our study represent more ubiquitous taxons which can be found in both animal and environmental sources. Of these, *Delftia* as well as Burkholderiaceae are commonly observed reagent contaminants and were highly abundant in the raw, non-decontaminated data, suggesting that they may represent persistent contamination in the reagents (Salter et al., 2014).

The microbial composition did not correlate between meconium and amniotic fluid apart from the apparent similarity at phylum level. The genera *Staphylococcus*, *Rubrobacter*, and *Clostridium* were relatively more abundant in meconium samples than in amniotic fluid samples. This is in contrast with some earlier studies in humans (Collado et al., 2016; He et al., 2020), but confirms the observations by Guzman et al. (2020) who also observed different microbial communities between the bovine meconium and amniotic fluid. As the fetus swallows the amniotic fluid, which is then concentrated and retained in the intestine as meconium, together with epithelial cells and intestinal secretions, it is unexpected that the two would harbor completely different bacteria. However, the amniotic fluid microbial signature may fluctuate dynamically over time, while meconium represents a more stable collection of substances accumulated over the gestation period. Moreover, fetal excretion into the amniotic fluid is limited in cattle. Meconium is usually expelled only after birth, and fetal urine largely accumulates in the allantoic cavity, between chorion and amnion, rather than in the amniotic cavity (Bongso and Basrur, 1976). Maternal microbial components may also be translocated via the placental and umbilical blood vessels directly to the fetal internal organs. Microbial macromolecules and even live bacteria may be transported by leukocytes via these blood vessels (Perez et al., 2007). In cattle, the less permeable synepitheliochorial placenta may also restrict the translocation of bacteria and their components from the dam to the fetus, in comparison to humans.

Bacteria were successfully cultured from 5 out of 24 meconium samples, representing both gram-negative and gram-positive bacterial genera known to live in mammalian hosts. This is in general agreement with the carefully controlled study by Guzman et al. (2020), who obtained rare bacterial colonies from the rumen of bovine fetuses, but the genera were different from our data. Of the bacteria observed in our study, *Fusobacteria* species are typically associated with mucous membranes, especially in the oral cavity, while *Cutibacterium* and *Kocuria* species have most often been isolated from the skin (Hofstad, 1992; Savini et al., 2010; Scholz and Kilian, 2016). Various *Acinetobacter* and *Streptococcus* species have physiological functions in their mammalian hosts, but the genera also include pathogenic and/or environmental species (Krzyściak et al., 2013; Touchon et al., 2014). Species of genera *Roseomonas* and *Achromobacter* are known to act as opportunistic pathogens but they have also been isolated from a wide variety of environmental sources (Reverdy et al., 1984; Rihs et al., 1993). One of our negative controls yielded *Kocuria* colonies. This was the only taxon shared between the actual meconium samples and the negative field controls, suggesting low-level contamination from the sampling environment.

All amniotic fluid samples were culture negative. Taken together, live bacteria appear extremely rare in healthy fetal cattle, in contrast to the prevalent bacterial DNA. This is not likely explained by limitations in culture methods, as we validated the growth conditions for calf intestinal core microbiota. The small number of viable bacteria observed in our study may be partially explained by fetal bacteriostatic substances. Both the mucosal surface of the intestine as well as the amniotic fluid contain lactoferrin and salivary scavenger and agglutinin (SALSA), which are able to suppress bacterial growth and viability (Reichhardt and Meri, 2016; Lisowska-Myjak et al., 2019). In human meconium, SALSA amounts to 10% of all proteins, highlighting its potential role in antimicrobial defense (Reichhardt et al., 2014). Individual differences in amounts of viable bacteria retrieved from meconium were also described in a recent study on dogs. Interestingly, puppies born without detectable meconium microbiota were shown to have a slower growth rate than those in which meconium microbiota were detected (Pipan et al., 2020).

In conclusion, we detected small amounts of diverse bacterial DNA and rare culturable bacteria in the meconium of full-term calves delivered by elective cesarean section. In the amniotic fluid, bacteria were not observed by 16S qPCR or culturing, but a microbial DNA profile was distinguishable from negative controls by deep sequencing. Based on these observations, bacterial components are translocated to the bovine fetus *in utero*, but the prenatal acquisition of live bacteria is likely not physiologically significant.

## Data Availability Statement

The dataset of the current study has been submitted to NCBI (https://www.ncbi.nlm.nih.gov) with accession PRJNA643145.

## Ethics Statement

The animal study was reviewed and approved by the Institutional ethics and animal welfare committee of the Faculty of Veterinary Medicine (EC2018/002—Ghent University, Belgium). Written informed consent was obtained from the owners for the participation of their animals in this study.

## Author Contributions

MN, LL, AI, and GO contributed to conception and design of the study. AH performed the bioinformatics, statistical analyses, and bacterial culture. AH and TG performed MALDI-TOF analyses. LL and JG organized the sampling and collected the samples. AH and LL wrote the manuscript. All authors contributed to manuscript preparation, read, and approved the submitted version.

## Conflict of Interest

The authors declare that the research was conducted in the absence of any commercial or financial relationships that could be construed as a potential conflict of interest.
