# Supplementary material for: The Composition of the Microbiota in the Full-Term Fetal Gut and Amniotic Fluid: A Bovine Cesarean Section Study
Source: Front Microbiol. 2021 Apr 30;12:626421. doi: 10.3389/fmicb.2021.626421 (PMC8119756; doi:10.3389/fmicb.2021.626421)
Supplement: Supplementary file 1 [file Presentation_1.pdf]

Supplementary material

1 Supplementary result

Supplementary Table 1. Most prevalent and abundant genera in the meconium and amniotic fluid samples.

Taxon	*Mec-Preva*	*Mec-Ave*	*Mec-SD*	*Amn-Preva*	*Amn-Ave*	*Amn-SD*
**Actinobacteria**	**100 %**	**9.3 %**	**5.3 %**	**100 %**	**9.3 %**	**5.7 %**
Corynebacterium 1	76 %	1.5 %	2.0 %	67 %	1.1 %	1.2 %
Rubrobacter	71 %	1.3 %	1.4 %	17 %	0.1 %	0.2 %
Cutibacterium	35 %	0.4 %	0.8 %	50 %	1.2 %	1.7 %
Kocuria	47 %	0.5 %	0.8 %	29 %	0.5 %	1.0 %
Mycobacterium	47 %	0.3 %	0.5 %	17 %	0.2 %	0.7 %
Nocardioides	47 %	0.5 %	0.8 %	33 %	0.3 %	0.5 %
Lawsonella	0 %	0.0 %	0.0 %	46 %	1.6 %	3.1 %
Micrococcus	41 %	0.6 %	1.2 %	29 %	0.5 %	1.3 %
**Bacteroidetes**	**100 %**	**12.3 %**	**7.9 %**	**96 %**	**10.2 %**	**8.5 %**
Bacteroides	53 %	2.1 %	3.1 %	25 %	0.9 %	2.8 %
Chryseobacterium	53 %	0.6 %	0.9 %	46 %	1.4 %	2.4 %
Cloacibacterium	29 %	1.3 %	4.4 %	42 %	2.0 %	5.3 %
Alistipes	41 %	0.5 %	0.6 %	0 %	0.0 %	0.0 %
Prevotellaceae UCG-003	41 %	0.6 %	1.0 %	0 %	0.0 %	0.0 %
Alloprevotella	35 %	0.5 %	0.7 %	13 %	0.3 %	0.8 %
Rikenellaceae RC9 gut group	35 %	0.8 %	1.4 %	21 %	0.2 %	0.6 %
**Firmicutes**	**100 %**	**30.0 %**	**16.5 %**	**100 %**	**34.0 %**	**16.2 %**
Streptococcus	59 %	2.5 %	6.1 %	88 %	6.0 %	5.5 %
Staphylococcus	76 %	4.2 %	4.6 %	83 %	6.4 %	5.8 %
Clostridium sensu stricto 1	71 %	3.7 %	8.6 %	29 %	0.7 %	1.4 %
Enterococcus	41 %	0.5 %	0.7 %	63 %	4.8 %	9.5 %
Bacillus	59 %	1.1 %	1.5 %	54 %	3.9 %	12.9 %
unclassified Lachnospiraceae	59 %	1.2 %	1.4 %	13 %	0.2 %	0.7 %
Christensenellaceae R-7 group	53 %	1.3 %	1.9 %	13 %	0.1 %	0.2 %
Lactobacillus	29 %	0.3 %	0.9 %	46 %	1.8 %	3.2 %
Ruminococcaceae UCG-010	41 %	0.5 %	0.7 %	4 %	0.2 %	0.8 %
Ruminococcaceae UCG-013	41 %	0.7 %	1.1 %	17 %	0.5 %	1.7 %
Ruminococcaceae UCG-014	41 %	0.6 %	0.9 %	8 %	0.1 %	0.2 %
[Ruminococcus] gauvreauii group	35 %	0.3 %	0.5 %	8 %	0.1 %	0.4 %
Acetitomaculum	35 %	0.3 %	0.5 %	4 %	0.0 %	0.1 %
Aerococcus	35 %	0.4 %	0.8 %	13 %	0.1 %	0.4 %
Family XIII AD3011 group	35 %	0.3 %	0.5 %	13 %	0.0 %	0.1 %
Mogibacterium	35 %	0.5 %	0.7 %	17 %	0.4 %	1.1 %
Ruminococcaceae UCG-005	35 %	0.6 %	1.2 %	21 %	0.5 %	1.2 %
Turicibacter	35 %	0.4 %	0.6 %	13 %	0.2 %	0.8 %

unclassified Peptostreptococcaceae	35 %	0.7 %	1.2 %	13 %	0.1 %	0.2 %
unclassified Ruminococcaceae	35 %	0.3 %	0.6 %	4 %	0.0 %	0.2 %
**Proteobacteria**	**100 %**	**44.4 %**	**18.9 %**	**100 %**	**43.0 %**	**14.8 %**
Delftia	94 %	11.8 %	10.8 %	92 %	5.0 %	3.6 %
Acinetobacter	88 %	2.7 %	2.3 %	71 %	3.1 %	3.0 %
unclassified Burkholderiaceae	82 %	2.1 %	2.5 %	58 %	1.1 %	1.6 %
Sphingomonas	47 %	0.9 %	1.5 %	71 %	5.0 %	6.3 %
Brevundimonas	71 %	0.9 %	1.2 %	38 %	0.6 %	1.4 %
Paracoccus	47 %	1.6 %	3.7 %	63 %	2.0 %	3.2 %
Massilia	59 %	2.8 %	7.4 %	29 %	0.5 %	1.1 %
Methylobacterium	59 %	0.9 %	1.0 %	46 %	0.7 %	1.1 %
Enhydrobacter	53 %	0.7 %	1.0 %	0 %	0.0 %	0.0 %
Pseudomonas	47 %	2.5 %	8.2 %	50 %	1.7 %	2.2 %
Psychrobacter	47 %	0.7 %	1.1 %	25 %	0.9 %	2.2 %
Stenotrophomonas	47 %	0.6 %	0.9 %	42 %	0.8 %	1.4 %
Cupriavidus	41 %	0.4 %	0.6 %	8 %	0.1 %	0.3 %
Novosphingobium	41 %	0.7 %	1.2 %	17 %	0.1 %	0.3 %
Alkanindiges	35 %	0.6 %	1.2 %	8 %	0.1 %	0.2 %
Allorhizobium-Neorhizobium-Pararhizobium-Rhizobium	35 %	0.5 %	0.9 %	17 %	0.2 %	0.7 %
Haemophilus	35 %	0.5 %	0.7 %	17 %	0.6 %	1.7 %
Rubellimicrobium	35 %	0.6 %	2.0 %	33 %	0.6 %	1.2 %
Sphingopyxis	35 %	0.3 %	0.8 %	21 %	0.2 %	0.5 %
Neisseria	29 %	0.4 %	1.0 %	33 %	0.7 %	1.4 %

2 Supplementary methods

2.1 Detailed description of the bioinformatics pipeline

2.1.1 Obtaining the data

The sequencing data for 69 samples was obtained from the sequencing lab in demultiplexed FASTQ format. The compressed tar.gz-package was uploaded to the now decommissioned supercluster *Taito* of Finnish Center for Scientific Computing (CSC). Package md5sum was checked before further decompression and analyses.

2.1.2. Quality check

FastQC 0.11.8 was ran for all the files and the resulting reports were checked manually[1]. FastQC reports were also compiled and assessed with MultiQC 1.7[2].

2.1.3 Trimming

All leftover primers and spacers were removed with Cutadapt v.1.10[3].

find -name "*R1_001.fastq.gz" -exec cutadapt -g CCTACGGGNGGCWGCAG -o '{}.TRIMMED_CUTADAPT_FW.gz' '{}' ';'

find -name "*R2_001.fastq.gz" -exec cutadapt -g GACTACHVGGGTATCTAATCC -o '{}.TRIMMED_CUTADAPT_REV.gz' '{}' ';'

The trimmed sequences were again checked with FastQC 0.11.8 and MultiQC 1.7[1,2].

2.1.4 Mapping file

A QIIME 2 compatible mapping file containing the sample metadata was created with Google Sheets and validated with Keemei[4,5].

2.1.5 QIIME2

Fastq.gz files were first imported to QIIME2 v2019.4 with command qiime tools import. The sequences were checked again using command qiime demux summarize[5]. The mean number of raw sequences was 114380 (max 187955, min 31, total 7892241) with read quality starting to drop below 20 in forward direction at 280 and reverse at 220. DADA2 was ran using the command dada2 denoise-paired with truncating option F280 and R220[10]. This results in an amplicon sequence variant (ASV) table.

Stats from the denoising and merging steps were checked with qiime metadata tabulate. Resulting data was explored with visual summaries created with qiime feature-table summarize and qiime feature-table tabulate-seqs.

The phylogenetic tree was created using qiime alignment mafft[6]. Highly variable positions adding noise were masked with qiime alignment mask. FastTree was used to create a phylogenetic tree from the masked alignment: qiime phylogeny fasttree and qiime phylogeny midpoint-root[7].

Preliminary diversity analysis was created with qiime diversity core-metrics-phylogenetic using sampling depth 15988.

The taxonomy was assigned according to the SILVA v132 QIIME release 99 %[8]. Representative set of sequences and taxonomy were imported to QIIME2 with qiime tools import. Reference reads were first extracted from the sequence directory with qiime feature-classifier extract-reads[9]. Then a Naïve Bayes classifier was trained to the curated taxonomy with qiime feature-classifier fit-classifier-naïve-bayes[10]. Finally, the actual classification was called with qiime feature-classifier classify-sklearn[11].

The taxonomy was visualised again with qiime metadata tabulate and qiime taxa barplot. After this the ASV table was exported from QIIME2 for handling in spreadsheet programs. First the table and taxonomy data was exported in biom-format with qiime tools export. The two were combined with biom package: biom add-metadata[13]. Finally the ASV table and table with taxonomy were converted to .tsv format with biom convert.

2.1.6 Data decontamination

Mitochondria, chloroplasts and singletons were filtered out, leaving only bacteria with at least phylum-level identification.

The processed data was filtered further to remove ASVs which represented probable contaminants[12]. An ASV was removed if its prevalence in actual samples was ≤2× its prevalence in field controls (empty sampling instruments exposed to the surgery room environment), and if its mean relative abundance in actual samples was ≤10× its mean abundance in field controls (Supplemental Figure 1). The filtering was performed separately for meconium and amnion samples. If less than 500 reads remained after the decontamination, as in six meconium samples, the sample was removed from further analyses.

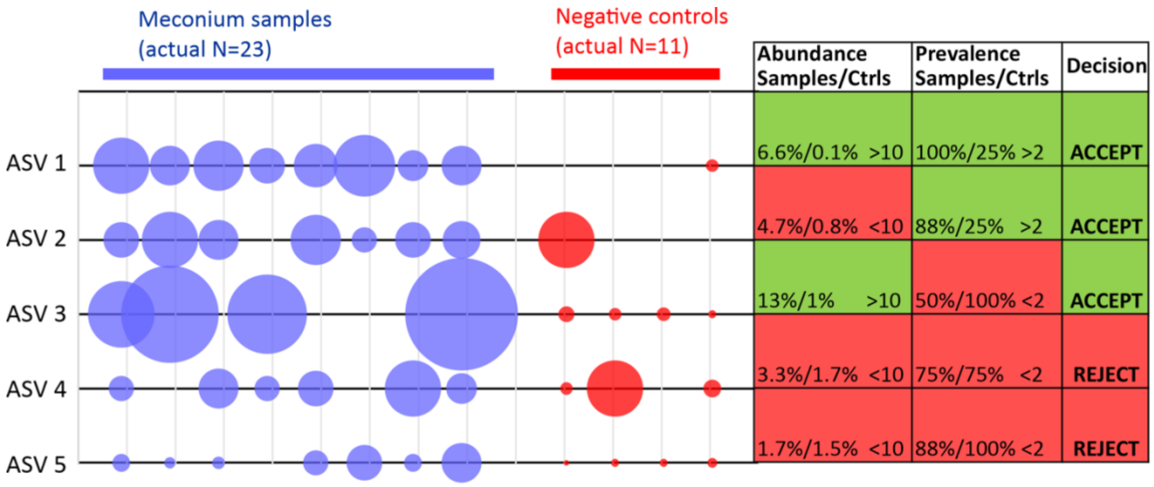

Supplementary Figure 1. Schematic illustration of the data decontamination strategy. ASV1 is accepted as the mean relative abundance and prevalence were both sufficiently high in meconium samples vs. negative controls. ASV1 was accepted as both abundance and prevalence were above threshold. ASV2 and ASV3 are accepted because either abundance or prevalence were sufficient. ASV4 and ASV5 are rejected as neither abundance or prevalence are above the threshold.

3 Supplementary references

1. Andrews, S. FastQC: a quality control tool for high throughput sequence data. (2011).
2. Ewels, P., Magnusson, M., Lundin, S. & Kaller, M. MultiQC: summarize analysis results for multiple tools and samples in a single report. *Bioinformatics* **32**, 3047–8 (2016).
3. Martin, M. Cutadapt removes adapter sequences from high-throughput sequencing reads. *EMBnet.journal* **17**, (2011).
4. Rideout, J. R. *et al.* Keemei: cloud-based validation of tabular bioinformatics file formats in Google Sheets. *Gigascience* **5**, 27 (2016).
5. Bolyen, E. *et al.* Reproducible, interactive, scalable and extensible microbiome data science using QIIME 2. *Nat. Biotechnol.* **37**, 852–857 (2019).
6. Katoh, K. MAFFT: a novel method for rapid multiple sequence alignment based on fast Fourier transform. *Nucleic Acids Res.* **30**, 3059–3066 (2002).
7. Price, M. N., Dehal, P. S. & Arkin, A. P. FastTree: computing large minimum evolution trees with profiles instead of a distance matrix. *Mol Biol Evol* **26**, 1641–50 (2009).
8. Quast, C. *et al.* The SILVA ribosomal RNA gene database project: improved data processing and web-based tools. *Nucleic Acids Res* **41**, D590-6 (2013).
9. Bokulich, N. A. *et al.* Optimizing taxonomic classification of marker-gene amplicon sequences

with QIIME 2’s q2-feature-classifier plugin. *Microbiome* **6**, 90 (2018).
10. Rennie, J. D., Shih, L., Teevan, J. & Karger, D. R. Tackling the poor assumptions of naive bayes text classifiers. in 616–623 (2003).
11. Pedregosa, F. *et al.* Scikit-learn: Machine learning in Python. **12**, 2825–2830 (2011).
12. Husso, A. *et al.* The composition of the perinatal intestinal microbiota in horse. *Sci. Rep.* **10**, 1–12 (2020).
13. McDonald, D. *et al.* The Biological Observation Matrix (BIOM) format or: how I learned to stop worrying and love the ome-ome. *Gigascience* **1**, 7 (2012).
